# Characterization of Retronasal Aroma Differences in High- and Low-Alcohol Jiangxiangxing Baijiu Using Temporal Sensory Evaluation, Retronasal Olfactory Thresholds, and Dose-over-Threshold Analysis

**DOI:** 10.3390/foods15071156

**Published:** 2026-03-29

**Authors:** Jun Zhang, Yehui Han, Xiaolong You, Xiaochun Zhu, Youlan Sun, Feng Hu, Xiaowei Yu, Shuang Chen, Yan Xu

**Affiliations:** 1 Laboratory of Brewing Microbiology and Applied Enzymology, State Key Laboratory of Food Science & Resources, Key Laboratory of Industrial Biotechnology of Ministry of Education, School of Biotechnology, Jiangnan University, 1800 Lihu Ave, Wuxi 214122, China; zjchem163@163.com (J.Z.); dblhui2@163.com (Y.H.); yxu@jiangnan.edu.cn (Y.X.); 2 Guizhou Xijiu Co., Ltd., Xishui 564622, China; youxiaolong1122@163.com (X.Y.); luqintian2022@163.com (X.Z.); 15761699434@163.com (Y.S.); hufeng2025xj@163.com (F.H.); 3 Key Laboratory of Quality and Safety of Jiangxiangxing Baijiu, State Administration for Market Regulation, Guiyang 550025, China

**Keywords:** low-alcohol, retronasal aroma, temporal sensory evaluation, retronasal olfactory thresholds, dose-over-threshold

## Abstract

Retronasal aroma strongly influences the flavor quality of low-alcohol Jiangxiangxing Baijiu. This study investigated the retronasal aroma differences between high- and low-alcohol samples (Sample-High and Sample-Low). Dynamic sensory evaluation revealed roasted, grain, floral and fruity, hay, and caramel notes were more pronounced in Sample-High, whereas Sample-Low exhibited stronger fatty and smoky notes, indicating insufficient retronasal aroma richness in Sample-Low. Analysis of 67 compounds showed no significant concentration differences, yet their retronasal olfactory thresholds varied markedly between alcoholic systems (ratio 1.04–4.75), leading to higher dose-over-threshold (DoT) values for several compounds in Sample-Low. Further analysis of the DoT distribution intervals showed Sample-High presented a more balanced distribution, whereas Sample-Low was strongly driven by several high-contribution compounds, which reduced the relative contributions of others. This unbalanced distribution may underlie the lower retronasal aroma richness observed in Sample-Low. These findings provide a new perspective for understanding retronasal aroma in low-alcohol Jiangxiangxing Baijiu.

## 1. Introduction

*Jiangxiangxing* Baijiu, as one of the important categories within the Chinese distilled liquor system, has received widespread attention owing to its distinctive and rich flavor characteristics. The formation of its characteristic style is closely associated with the numerous flavor compounds generated during its unique brewing process, which collectively shape its sensory profile and further influence consumer perception of overall quality and typicity [1,2,3]. Aroma, as one of the important components of the overall flavor of *Jiangxiangxing* Baijiu, shapes the initial olfactory impression of consumers and plays a critical role in their perception of product quality and preference. As consumer preferences diversify, the market for lower-alcohol Baijiu has shown steady growth, accompanied by heightened expectations for aroma quality. Against this background, clarifying how alcohol concentration influences the olfactory characteristics of Baijiu has become a central scientific topic of shared concern for both academia and industry.

Current studies in Baijiu flavor chemistry have mainly concentrated on identifying aroma compounds and assessing their contributions to overall sensory perception. These studies have predominantly focused on high-alcohol Baijiu (typically ≥50% vol) and have been limited to the orthonasal olfactory pathway. However, human olfaction involves both orthonasal and retronasal pathways [4,5]. Compared with orthonasal perception, retronasal olfaction provides a more accurate representation of the dynamic evolution of aroma during the consumption of Baijiu, thereby playing a pivotal role in defining the overall sensory experience. Nevertheless, systematic studies on the retronasal olfactory characteristics of Baijiu remain scarce. Among the few available studies, some have concentrated exclusively on single-alcohol systems [6], whereas others have lacked an integrated framework linking alcohol content variation with the functional roles of aroma perception [7,8]. To date, the influence of alcohol concentration on the dynamic evolution of retronasal aroma in Baijiu remains poorly elucidated, revealing a distinct knowledge gap in Baijiu flavor research. Notably, the perceptual differences between high- and low-alcohol *Jiangxiangxing* Baijiu are closely associated with variations in the differential contributions of key aroma compounds. From the perspective of flavor chemistry, the potential retronasal impact of individual compounds can be quantitatively assessed using the dose-over-threshold (DoT). Among relevant parameters, the retronasal olfactory threshold (ROT) serves as a key indicator for quantitative evaluation, indirectly reflecting the intrinsic capacity of individual compounds to contribute to retronasal aroma. However, research on the ROT of key aroma compounds in Baijiu remains considerably limited. First, existing data are primarily based on single-alcohol systems [6,9]. Second, the number of compounds with established ROT values is insufficient for comprehensive comparison. Critically, no systematic studies have yet explored how ethanol concentration influences the ROT of key aroma compounds. These limitations obscure the chemical basis of post-ingestive retronasal differences between high- and low-alcohol *Jiangxiangxing* Baijiu and hamper flavor optimization of low-alcohol products. Therefore, clarifying the ROT and DoT of key aroma compounds across different alcohol levels can bridge the existing research gap and provide essential theoretical and practical insights into the chemical basis of retronasal sensory differences among Baijiu with different alcohol levels.

Therefore, to clarify the retronasal aroma differences between high- and low-alcohol *Jiangxiangxing* Baijiu, this study aimed to: (1) characterize these differences across different stages using temporal sensory evaluation methods; (2) determine the ROT of selected aroma compounds in 40% vol and 50% vol ethanol aqueous solution systems; and (3) elucidate the chemical basis of these retronasal aroma differences by integrating compound DoT values across high- and low-alcohol systems with the differential patterns of potential aroma contribution distributions within each system. This study establishes a theoretical foundation for quality improvement and precise flavor modulation in low-alcohol *Jiangxiangxing* Baijiu, highlighting both its academic significance and practical relevance.

## 2. Materials and Methods

### 2.1. Baijiu Samples

Given the limited availability of commercially available low-alcohol *Jiangxiangxing* Baijiu products, 39% vol and 53% vol were selected as practical comparison points to provide a clearer contrast between typical low- and high-alcohol products on the market. Two *Jiangxiangxing* Baijiu samples from a distillery in Southwest China were therefore used in this study: a low-alcohol sample (39% vol, labeled Sample-Low) and a high-alcohol sample (53% vol, labeled Sample-High). Although some co-authors are affiliated with the company, it provided only access to analytical instruments and technical personnel, and had no involvement in the selection of the samples or alcohol levels. Both products have been marketed for years and thus show a certain degree of market representativeness in flavor profile. Samples were stored at 20 °C in a temperature-controlled room throughout the experiment.

### 2.2. Reagents and Chemicals

All compounds used in the sensory experiments were food-grade, with detailed provided in Table S1. Food-grade ethanol (95% vol) was supplied by Shanghai Titan Co., Ltd. (Shanghai, China), and purified water was obtained from Hangzhou Wahaha Group Co., Ltd. (Hangzhou, China).

### 2.3. Sensory Analysis

#### 2.3.1. Sensory Panel

Referring to a previous study [10], a sensory panel consisting of 36 assessors had already been established. Based on this panel, 23 assessors (13 males and 10 females, aged 25–30 years) were pre-selected as the core candidate semi-trained panel for the present study.

All were current graduate students from Jiangnan University (Wuxi, China) with over two years of experience in Baijiu sensory evaluation. In addition, six senior certified Baijiu tasters from renowned domestic distilleries, each possessing over ten years of sensory experience, were invited to provide expert guidance, participate in descriptor discussion, and assist in the performance evaluation of the semi-trained panelists. These tasters were not included in the formal sensory dataset used for statistical analysis. All candidate semi-trained panelists and senior tasters were in good health, had normal olfactory function, and no history of alcohol allergy.

Before the formal experiment, the training program was refined based on prior curricula, and a short-term professional course consisting of three two-hour sessions was conducted, focusing on the identification of sensory attributes and the establishment of reference standards (Table S2). To avoid potential bias from prior knowledge, semi-trained members were not informed of the chemical composition of each reference during training. Moreover, some high- and low-alcohol Jiangxiangxing Baijiu samples with typical aroma characteristics were used to strengthen their ability to identify these sensory features under varying ethanol concentrations. Following the training, the 23 semi-trained panelists underwent a comprehensive performance evaluation and were confirmed as qualified for the formal sensory experiments. Written informed consent was obtained from all participants prior to the study, and appropriate remuneration was provided upon completion. The study was reviewed and approved by the Human Research Ethics Committee of Jiangnan University.

The formal sensory evaluation was conducted in the professional sensory laboratory of the Synergistic Innovation Centre at Jiangnan University. The testing environment was maintained at 25 ± 1 °C, with clean and odor-free air. The panelists performed evaluations in individual sensory booths while wearing nose clips before sample intake. Each sample (2 mL) was accurately measured and served in standardized tasting glasses labeled with random three-digit codes, which were presented in a randomized order. To verify within-panelist consistency and sample repeatability, the same Baijiu sample (identified by different random codes) was presented three times during the evaluation sequence. After completing each set of samples, panelists rinsed their mouths with an aqueous ethanol solution of about 3% vol for approximately 10 s, followed by thorough rinsing with purified water at room temperature. The entire rinsing procedure lasted about 1 min, after which a mandatory rest period of at least 10 min was required before evaluating the next sample.

#### 2.3.2. Retronasal Dual-Mode Temporal Check-All-That-Apply (RDM-TCATA) Analysis

The development of dynamic sensory analysis techniques has greatly advanced the characterization of the temporal evolution of sensory attributes during consumption. While the check-all-that-apply method allows for comprehensive collection of retronasal aroma descriptors, it fails to capture their temporal evolution. In contrast, temporal check-all-that-apply integrates the time dimension, enabling dynamic tracking of aroma evolution [11]. Given the inherent complexity and temporal variability of retronasal aroma perception during Baijiu consumption, this study developed an innovative dual-mode system, RDM-TCATA, to capture retronasal aroma attributes and their temporal citation dynamics under different evaluation modes.

To establish descriptors scientifically aligned with the retronasal aroma characteristics of Sample-High and Sample-Low, six senior certified Baijiu tasters participated in on-site evaluations and discussions based on actual samples. Through consensus among all panelists, a final predefined list of retronasal aroma descriptors was developed and later applied in the RDM-TCATA experiments. Following previous studies [12,13], this study also considered three evaluation intervals, attack (A, 0–15 s), evolution (E, 16–60 s), and finish (F, 61–150 s), to investigate the dynamic changes in retronasal aroma perception across different post-ingestion stages in samples. Prior to the experiment, panelists received supplementary training to standardize descriptor definitions and software operation procedures, thereby ensuring consistency in evaluation criteria. The experiment was conducted in two consecutive stages. In the first stage (Mode 1), panelists used specialized software to record retronasal aroma descriptors. Within 150 s after swallowing each sample, they selected and logged perceived retronasal aroma descriptors in real time from a predefined descriptor list. After completing all evaluations, the citation proportion for each attribute was calculated as the percentage of its selections relative to the total number of records within a specific time segment. This metric was used to characterize the perceived frequency of each attribute across different stages and to identify representative retronasal descriptors for further analysis. In the second stage, the descriptors selected during the first stage were used as evaluation indicators for the Mode 2 experiment. During this process, panelists continuously recorded their perceived retronasal aroma attributes in real time through specialized software, enabling the generation of time–citation proportion curves for each sensory attribute. Each sample was evaluated in triplicate by every panelist to ensure data reliability.

#### 2.3.3. Retronasal Dual-Attribute Time–Intensity (RDA-TI) Analysis

From the perspective of temporal changes in sensory intensity, traditional time–intensity methods effectively capture the evolution of a single attribute over time but are limited by their time-consuming procedures [14]. As an advanced extension, dual-attribute time–intensity improves the efficiency of temporal sensory data collection [15]. The RDA–TI method developed in this study enabled real-time monitoring of dynamic changes in retronasal aroma intensity, providing a quantitative approach to compare the temporal evolution of sensory intensity between high- and low-alcohol samples. Before the experiment, six experienced tasters conducted multiple rounds of on-site discussions and sensory validations using experimental samples. Based on these sessions and the initial perceptual data distributions obtained from the pre-experiment sensory panel, intensity grading standards and corresponding value ranges for each key retronasal aroma descriptor were systematically established. To account for differences in perceived intensity levels among various aroma attributes, attribute-specific maximum intensity thresholds were defined, and values exceeding these limits were fixed in the software. Panelists were instructed to rate intensities strictly within the range of 0 to the respective threshold, thereby standardizing the evaluation scale and minimizing scoring variability caused by individual differences in maximum intensity perception. During data acquisition, the system operated with a 2 s refresh cycle. After swallowing each sample, panelists continuously rated the aroma intensities of two randomly paired attributes in real time on the evaluation scale. After data acquisition, key parameters were extracted from the TI curves, including the time to reach maximum intensity (T_max_), maximum intensity (I_max_), total duration (T_tot_), area under the curve (AUC), duration of the 90% maximum intensity plateau (Plateau_90%_), and the decay time constant after reaching peak intensity (τ). These parameters were used mainly to characterize and compare the dynamic retronasal aroma patterns of high- and low-alcohol samples under different temporal dimensions. Previous studies have shown that the temporal decay of retronasal aroma attributes after reaching maximum intensity can be approximated by an exponential function [16,17,18]. Accordingly, based on this pattern, the post-peak intensity decay of each retronasal aroma attribute was fitted using an exponential model (Equation (1)). The τ was subsequently derived from the fitted model (Equation (2)), where a larger value indicates stronger temporal persistence of the corresponding aroma attribute. In Equation (1), *y* denotes the perceived aroma intensity, *x* represents the time variable, *a* corresponds to the coefficient related to the initial intensity, *b* indicates the decay rate constant, and *c* defines the time offset.

(1) $${y=a\cdot e}^{b\cdot (x-c)}$$

(2) $$\tau =\frac{1}{\left|b\right|}$$

### 2.4. Determination of Concentrations of Representative Compounds in Samples

Given the large number of aroma compounds previously identified in high- and low-alcohol *Jiangxiangxing* Baijiu, a total of 67 representative compounds from 11 chemical categories were selected for detailed analysis. The selection was based on a combination of considerations, including relatively high concentrations, significant odor activity values, distinctive physicochemical properties, and the ability of the compounds to represent the major aroma categories. It should be noted that conventional odor activity values are derived from orthonasal olfactory thresholds and were therefore used in this study only as a preliminary screening criterion, rather than as a decisive indicator of retronasal relevance. Accordingly, although some compounds with limited orthonasal prominence but potentially greater retronasal importance may not have been fully captured, this strategy still allowed broad coverage of the major aroma categories while maintaining experimental feasibility. This selection strategy was similar to approaches reported in the literature [19], with only minor differences. The quantification of aroma compounds in this study was based on a multi-technique analytical framework established in our previous studies [10,20]. Specifically, direct injection coupled with gas chromatography–flame ionization detection was used for volatile abundant components; dispersive liquid–liquid microextraction coupled with gas chromatography–mass spectrometry was used for volatile phenols and acidics; headspace solid-phase microextraction coupled with comprehensive two-dimensional gas chromatography–time-of-flight mass spectrometry was used for volatile trace components; headspace solid-phase microextraction coupled with gas chromatography–pulsed flame photometric detection and solid-phase extraction coupled with ultra-performance liquid chromatography–tandem mass spectrometry were used for major volatile sulfur compounds; and 2,4-dinitrophenylhydrazine derivatization coupled with ultra-performance liquid chromatography–tandem mass spectrometry was used for volatile aldehydes and ketones. In this work, only the specific operations for the analysis of furans and lactones were supplemented, employing dispersive liquid–liquid microextraction coupled with gas chromatography–tandem mass spectrometry to further enhance the overall quantitative analytical framework.

A total of 4 mL of Baijiu sample was mixed with 16 mL of saturated sodium chloride solution and 4 mL of an ethyl acetate–acetone mixture (1:1, *v*/*v*) in a 50 mL glass vial. Subsequently, 20 μL of the internal standard solution (guaiacol-d3, 100 mg/L) was added. The mixture was vortexed at 500 rpm for 10 min to ensure efficient extraction, and then allowed to stand for 10 min. The upper organic phase was collected, dehydrated with anhydrous sodium sulfate, and concentrated under a gentle stream of nitrogen to approximately 250 μL for instrumental analysis. The concentrated extract was analyzed using a gas chromatograph coupled with a triple quadrupole mass spectrometer (8890 GC–7000E MS/MS, Agilent Technologies, Santa Clara, CA, USA) equipped with a DB-FFAP capillary column (60 m × 0.32 mm × 0.25 μm; Agilent Technologies, USA). High-purity helium (≥99.999%) was used as both the carrier gas (1.4 mL/min) and quenching gas (2.25 mL/min), while nitrogen served as the collision gas (1.5 mL/min). The injector temperature was maintained at 250 °C, and 1 μL of the concentrated extract was injected in splitless mode with a solvent delay of 8 min. The oven temperature program was as follows: initial temperature of 40 °C held for 2 min, ramped at 4 °C/min to 150 °C and held for 2 min, then increased at 6 °C/min to 230 °C and held for 15 min. The ion source, transfer line, and quadrupoles 1 and 2 were maintained at 230 °C, 250 °C, 150 °C, and 150 °C, respectively. Mass spectrometric detection was performed in electron ionization (EI, 70 eV) mode using dynamic multiple reaction monitoring (dMRM) for data acquisition. For method development, target compounds were initially analyzed in full-scan mode to determine their retention times and precursor ions, with the most intense precursor–product ion pairs selected based on signal abundance. Product ion scans were subsequently performed at a collision energy of 10 eV to identify the most responsive fragment ions. Subsequently, the MRM parameters were automatically optimized over a collision energy range of 0–25 eV using MassHunter Workstation software (v12.1, Agilent, Palo Alto, CA, USA). The two transitions exhibiting the highest signal intensities and their corresponding optimal collision energies were selected for quantification. The MS/MS parameters and quantitative calibration curves for all compounds were summarized in Table S3.

### 2.5. Determination of ROT of Representative Compounds in High- and Low-Alcohol Systems

As emphasized in the Introduction, the determination of ROT is critically important. Accordingly, the ROTs of 67 aroma compounds were systematically measured using the three-alternative forced-choice method according to ISO 13301:2018 [21]. Food-grade compounds were dissolved in anhydrous ethanol to prepare initial stock solutions, ensuring complete solubility. Preliminary experiments were conducted to determine the detectable concentration range of each compound in the target ethanol aqueous solution systems (40% or 50% vol). Based on these results, an intermediate concentration was selected as the reference point, and six concentration levels were prepared using a three-fold serial dilution. The 40% vol and 50% vol ethanol aqueous solution systems were used here as standardized reference conditions for controlled threshold determination and comparative screening of compounds with potential retronasal relevance at two representative alcohol levels, rather than to fully reproduce the real Baijiu matrix. The use of whole-number alcohol levels also facilitates comparison with future threshold studies and improves cross-study comparability. Each gradient sample was paired with a blank solution of 40% vol or 50% vol to form a test set, with 2 mL of each sample dispensed into coded cups labeled with random three-digit numbers. During the formal experiment, panelists wore nose clips before taking each sample and removed them only after the sample was placed in the mouth. Throughout the test, samples were evaluated in ascending order of concentration to prevent interference from residual high-intensity stimuli. Specifically, panelists were instructed to take the sample into their mouths within 0–2 s and distribute it evenly. They then kept their mouths closed and breathed naturally, spitting out the sample at the 5 s. Between 5 and 10 s, panelists focused on perceiving aromas through the retronasal pathway and identified the sample containing the target compound. To minimize sensory fatigue and adaptation, panelists rested for 10 min after rinsing their mouths, as previously described, before evaluating the next set of samples. Finally, the geometric mean of the individual best-estimate thresholds obtained from the 23 qualified semi-trained panelists was calculated and used as the final ROT for each compound under the specified alcohol concentration.

### 2.6. Statistical Analysis

The relative standard deviations of compound concentrations in different samples were evaluated. Cluster analysis and heatmap visualization of different aroma descriptors were performed using Tbtools (v2.450) [22]. Differences in elicitation proportions between the two samples were assessed in XLSTAT (version 2019.2.2, Addinsoft, Paris, France) using Fisher’s test at a significance level of 5%. The key parameters extracted from the TI curves were analyzed using DPS software (Version 9.01, Hangzhou, China) by one-way analysis of variance followed by Duncan’s multiple range test. Horizontal bar charts were generated on the NGplot platform [23]. Subsequently, a two-tailed Welch’s *t*-test was conducted to assess the significance of DoT differences for individual compounds between the two alcohol-level systems.

## 3. Results and Discussion

### 3.1. Analysis of Time–Citation Proportion Differences in High- and Low-Alcohol Systems

**Figure 1:**
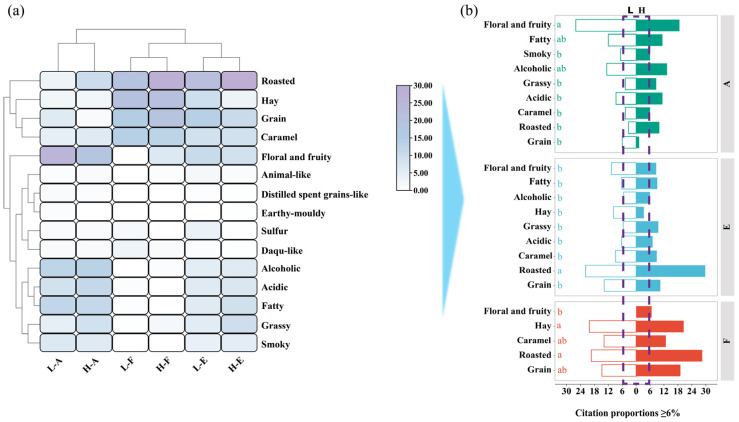
Differences in retronasal aroma descriptors between Sample-Low and Sample-High across stages A, E, and F. In both (**a**,**b**), L represents Sample-Low, H represents Sample-High, while A, E, and F correspond to the attack phase (0–15 s), evolution phase (16–60 s), and finish phase (61–150 s), respectively. Lowercase letters (a, b.) indicated significant differences among groups (*p* < 0.05).

Based on the analysis of Model 1, the distribution of retronasal aroma descriptors for Sample-Low and Sample-High across stages A, E, and F was clearly defined (Figure 1a). From the citation frequencies of aroma attributes, ten main retronasal aroma descriptors with relatively high citation rates were identified, including grain, floral and fruity, acidic, alcoholic, roasted, hay, caramel, smoky, fatty, and grassy notes. The total number of selected attributes was consistent with recommended guidelines [24]. Overall, these descriptors broadly correspond to the major aroma dimensions reported in previous studies on the characteristic sensory profile of Jiangxiangxing Baijiu [25]. According to the data distribution, a 6% citation threshold was applied to determine the dominant retronasal aroma attributes most frequently reported by panelists over time (Figure 1b). This threshold was based on the overall distribution of citation frequencies and those observed in the F phase. While effective, it was an empirical decision. Results indicated that during the A phase, eight notes in Sample-High exceeded the threshold, whereas only six were observed in Sample-Low, suggesting a greater diversity of retronasal aroma perception in the initial stage of Sample-High. In the E phase, the number of frequently cited notes was similar between the two samples, with eight notes identified in Sample-High and seven in Sample-Low. In the F phase, the overall citation frequency of notes decreased, yet Sample-High still maintained five notes above the threshold (floral and fruity, roasted, caramel, hay, and grain), while Sample-Low retained four (roasted, caramel, hay, and grain). This finding suggested that Sample-High maintained a higher level of aromatic diversity in the lingering stage of retronasal perception. However, the current results only reflect the frequency of attribute citations, without considering their time–citation proportions within the perceptual process.

**Figure 2:**
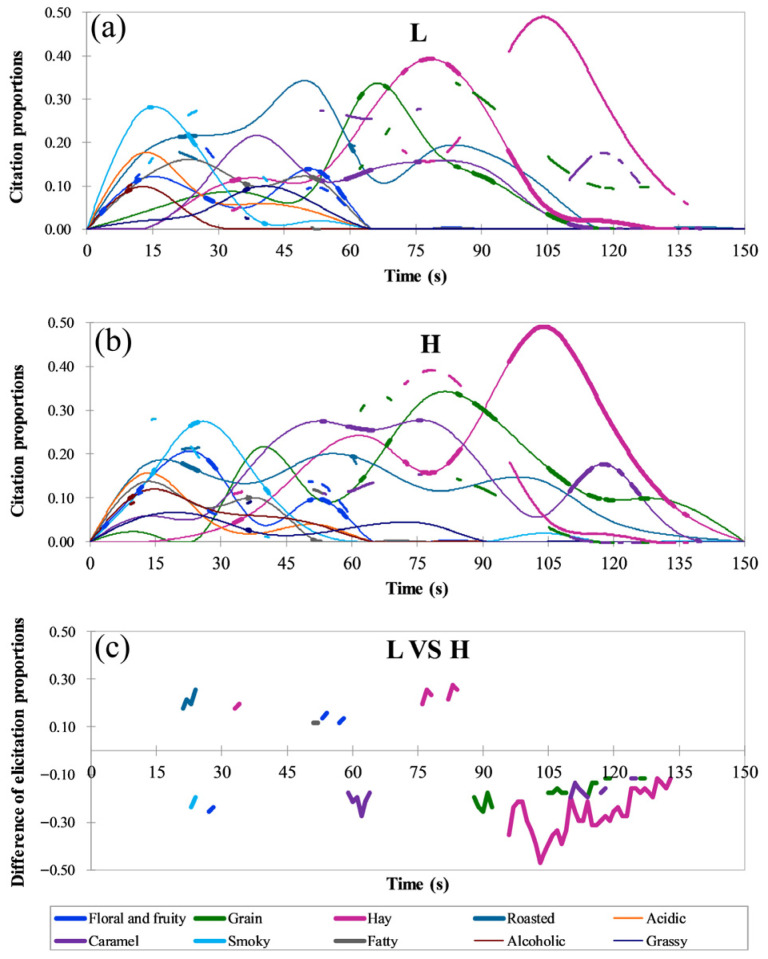
Differences in the time–citation proportions of important retronasal attributes between Sample-Low and Sample-High. Here, L represents Sample-Low and H represents Sample-High. When key reference curves are displayed, the corresponding attribute curves are shown in bold. Panels (**a**–**c**) represent the time–citation proportion curves of different retronasal attributes for Sample-Low, Sample-High, and the differences in elicitation proportions between the two samples, respectively. Differences in elicitation proportions were assessed using Fisher’s test (*p* < 0.05) and interpreted mainly in the context of the overall temporal evolution pattern of each attribute.

A Model 2 experiment was further conducted to analyze the temporal dynamics of the time–citation proportions for each note (Figure 2). Results showed that during the A phase, the citation proportions of both Sample L and Sample H gradually increased, indicating that retronasal aroma perception developed rapidly during the initial swallowing phase. In contrast, Sample H showed stronger simultaneous enhancement and interaction among multiple attributes, exhibiting a multidimensional release pattern. Sample L was dominated by the smoky note, with weaker contributions from other aromas, reflecting limited overall aroma synergy. This stage underscored the distinct differences between the two samples in their aroma release rhythm and coordination patterns, although no clear difference was detected in their overall citation proportions. Stage E represented the critical phase of aroma evolution, during which the differences between the two samples became more pronounced. The dominant sensory profile of Sample-High featured smoky, floral and fruity, roasted, caramel, grain, and hay notes, creating a complex and well-layered aromatic structure. In contrast, Sample L was initially dominated by the smoky note, which was rapidly replaced by roasted and caramel notes, followed by a gradual increase in the citation proportions of hay and grain notes in the later stages. Significance analysis (*p* < 0.05, Figure 2c) showed that during the early phase (20–30 s), smoky and floral and fruity notes were more pronounced in Sample-High, while the roasted note prevailed in Sample-Low. In the middle phase (30–40 s), the hay note became more pronounced in Sample-Low. In the later phase (50–60 s), fatty and floral and fruity notes became more perceptible in Sample-Low, whereas caramel notes in Sample-High continued to increase steadily. During the F phase, corresponding to the decline of retronasal aroma perception, time–citation proportions exhibit an overall downward trend. In Sample-High, hay, caramel, grain, and roasted notes remained consistently dominant during the later stage. In contrast, Sample-Low showed greater fluctuations in aroma dominance. Although hay and grain notes were briefly dominant, they faded quickly, leaving a simpler and less persistent afterodor. The significance difference curves (Figure 2c) revealed notable differences between the two samples in caramel, hay, and grain notes (*p* < 0.05), with Sample-High exhibiting a richer and more persistent perceptual profile.

In summary, the RDM-TCATA analysis indicated that Sample-High exhibited a more pronounced advantage in retronasal aroma performance, aligning with previous reports of enhanced aroma quality in high-alcohol wines [26]. Furthermore, these results indicate that ethanol content plays a key role in determining the richness and temporal persistence of retronasal aromas. Similarly, studies on rum have shown that ethanol concentration strongly affects its temporal sensory dynamics, underscoring the central role of ethanol in shaping the time-dependent perception of distilled spirits [27]. In this study, the RDM-TCATA results were used primarily to identify retronasal attributes showing differentiated temporal patterns between the two samples. Accordingly, the differences are better understood at the level of overall temporal evolution rather than isolated time points, and individual significant findings should therefore be interpreted with caution. In addition, it should be acknowledged that the RDM–TCATA approach has limited capacity to capture changes in perceived aroma intensity. Accordingly, a time–intensity experiment was subsequently conducted to further quantify the dynamic changes in retronasal notes.

### 3.2. Analysis of Time–Intensity Differences in High- and Low-Alcohol Systems

**Figure 3:**
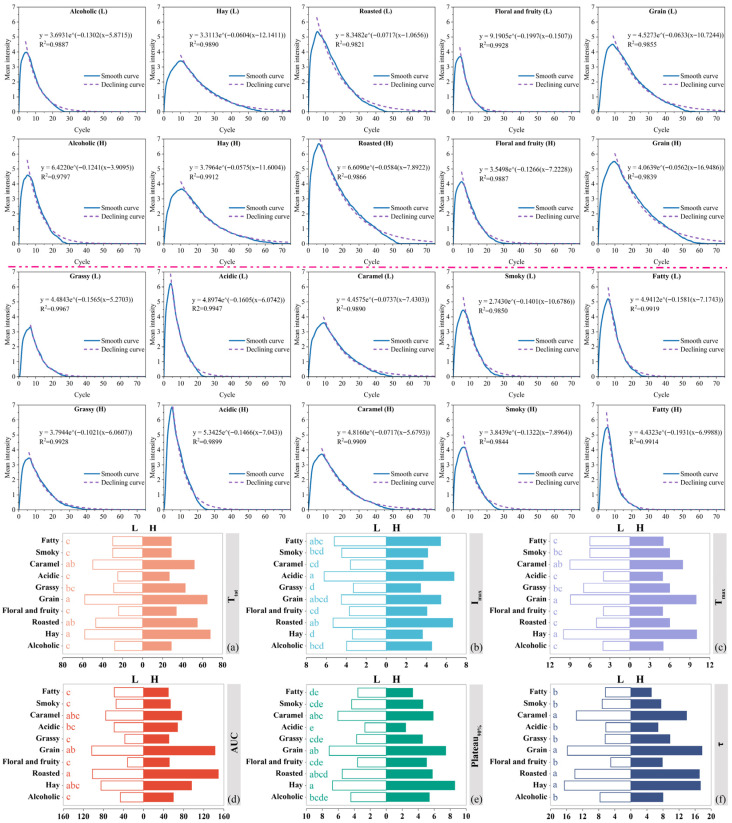
Time–intensity fitting curves and differences in key parameters of important retronasal notes between Sample-Low and Sample-High. Here, L represents Sample-Low and H represents Sample-High. Subfigures (**a**), (**b**), (**c**), (**d**), (**e**), and (**f**) represent the intensity parameters T_tot_, I_max_, T_max_, AUC, Plateau_90%_, and τ, respectively. Lowercase letters (a, b, c, etc.) next to aroma attributes indicate the grouping of between-sample differences under the corresponding TI parameter. Earlier letters (a > b > c > d > e) indicate relatively larger between-sample differences.

Based on the time–intensity sensory evaluation, the quantitative results offered a clearer representation of the differences between Sample-High and Sample-Low in aroma intensity, duration, and decay behavior (Figure 3). The T_tot_ data (Figure 3a) revealed that the total durations of hay, grain, roasted, and caramel notes in Sample-High each exceeded 50 cycles, whereas only three notes in Sample-Low reached this level, and six had values below 30 cycles. Notably, the fruit and floral note exhibited the shortest duration, suggesting that higher ethanol concentrations markedly suppressed its perception, a trend consistent with previous research findings [28]. Moreover, reducing alcohol content has been reported to diminish the perceived persistence of wine in the mouth [29], whereas a higher ethanol content prolongs the finish duration [30]. Consequently, the greater proportion of short-lived aromas in Sample-Low likely accelerated flavor decay, explaining its weaker and shorter retronasal impression.

The I_max_ and T_max_ data demonstrated that Sample-High exhibited a distinct advantage in some notes (Figure 3b,c). Notably, the roasted, grain, alcoholic, floral and fruity, acidic, and hay notes exhibited both higher I_max_ values and longer T_max_ durations in Sample-High. For instance, the roasted and grain notes showed over a 20% increase in I_max_, with their T_max_ extended by nearly one cycle. This indicated that these notes in Sample-High not only reached higher perceived intensity but also sustained their peak perception longer, thereby enhancing the persistence and complexity of retronasal aroma perception. In contrast, Sample-Low exhibited lower I_max_ values, with only the smoky note showing a slight increase. Except for grassy, fatty, and caramel notes, the T_max_ values of all others occurred earlier than those in Sample-High. Thus, compared with the low-alcohol sample, the high-alcohol sample showed greater aroma intensity and a later peak perception, suggesting a richer and longer-lasting retronasal experience under the present sample conditions.

AUC, an integrated index of intensity and duration, further confirmed these differences. Previous studies reported that high-alcohol samples could extend the T_tot_ of certain aroma features, leading to larger AUC values [31]. Accordingly, Figure 3d consistently showed that, except for fatty and smoky notes, all others in Sample-High displayed higher AUC than those in Sample-Low, with increases exceeding 37% for floral and fruity, roasted, grain, and grassy notes. Notably, the notes of roasted and grain not only remained longer but also accumulated to higher perceived intensities, further highlighting the superiority of Sample-High in retronasal aroma longevity and complexity.

The Plateau_90%_ data indicated that Sample-High maintained higher intensities for most key aroma attributes over a longer duration (Figure 3e). The intensities of floral and fruity, hay, grassy, and alcoholic notes increased by more than 20%, while the persistence of negative attributes, such as fatty, was reduced. In contrast, Sample-Low exhibited only slight advantages in fatty, acidic, and caramel notes. These findings further confirmed the overall superiority of Sample-High in aroma stability and sensory quality.

The τ data further revealed that the decay of some notes in Sample-High occurred more gradually than in Sample-Low (Figure 3f). Specifically, the τ values of grain, hay, and roasted exceeded 17, while floral and fruity as well as grassy notes increased by more than 50% compared to Sample-Low. These results suggested that higher ethanol content slowed the decline of aroma intensity after its peak and enhanced the persistence and complexity of retronasal aroma. In contrast, Sample-Low exhibited a faster decline in several notes, with only the fatty note presenting a higher τ value, which may negatively affect the perception of retronasal aroma. The distinct trends observed across different aroma characteristics suggested that aroma persistence may be associated with the structural properties of the corresponding compounds [32]. Furthermore, these findings reinforce the important role of ethanol in modulating the persistence of retronasal aroma [33].

Analysis of the differences in intensity parameters (I_max_, T_max_, Plateau_90%_, T_tot_, AUC, and τ) revealed that roasted, grain, floral and fruity, hay, and caramel notes were more prominent in Sample-High, whereas fatty and smoky notes dominated in Sample-Low. These differences indicate that most notes in Sample-High exhibited greater intensity, longer persistence, and richer cumulative perception, reflecting its overall advantage in retronasal aroma performance. This TI-based interpretation is better understood in terms of the integrated pattern across multiple temporal dimensions rather than any single parameter alone. Comparisons within each parameter primarily reflected the relative magnitude of between-sample differences among aroma attributes, and should be interpreted with caution because no additional global multiple-comparison correction was applied.

### 3.3. Analysis of Retronasal Aroma Differences Based on Compound DoT in High- and Low-Alcohol Systems

**Figure 4:**
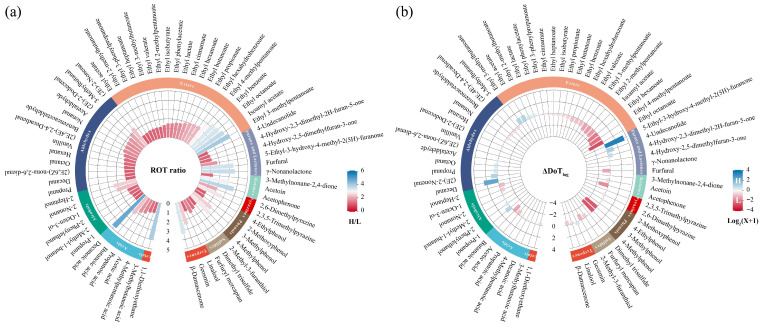
Differences in ROT and DoTlog values among various classes of aroma compounds between the high- and low-alcohol systems. (**a**) ROT ratios of different classes of compounds in 40% vol and 50% vol ethanol aqueous solutions; (**b**) logarithmic differences in DoT values of individual compounds between high- and low-alcohol systems.

To investigate how ethanol concentration influences retronasal aroma perception in Baijiu, this study systematically evaluated the ROT and DoT of 67 compounds in high- and low-alcohol systems, as summarized in Table 1. From the perspective of compound concentration, the overall difference between the two samples was consistently minimal. The concentrations of 52 compounds varied by only 1- to 2-fold, which suggested that these changes were unlikely to be the main factor underlying the marked difference in retronasal aroma detected between the samples. In contrast, the ROT results revealed pronounced differences among compounds from different categories in response to ethanol concentration. For most compounds, ROT values in the low-alcohol system were substantially lower than those in the high-alcohol system, with ROT ratios ranging from 1.04 to 4.75, as illustrated in Figure 4a. These findings indicated that the effect of ethanol on the release of compounds with different structures was not uniform, and that more compounds in the low-alcohol system were likely to be detected at lower concentrations. However, whether this trend resulted in a richer retronasal aroma experience was further examined. The DoT of each compound was calculated from its concentration and corresponding ROT value, as summarized in Table 1. It should be noted that DoT is better regarded as a potential indicator whose primary value lies in identifying compounds with relatively high potential retronasal aroma contribution, rather than in precisely quantifying sensory contribution in the real sample matrix. Because the ROTs were obtained in simplified ethanol aqueous solution systems at 40% vol and 50% vol, rather than in systems exactly matching the two actual Baijiu samples, the resulting DoTs are more appropriately interpreted as approximate comparative measures. Although their absolute values may involve some deviation, they remain informative for compound screening, relative comparison, and trend assessment.

To reduce the impact of extreme DoT values on the interpretation of overall trends, the original DoT data were log-transformed (DoT_log_) to decrease high-value variability and enhance the visibility of mid- to low-value ranges, thereby improving the comparability of potential aroma contributions between the two systems. Based on these calculations, the logarithmic differences between the two systems were expressed as ΔDoT_log_. As shown in Figure 4b, most compounds had ΔDoT_log_ below 0, which suggested that Sample-Low likely possessed a higher potential retronasal aroma contribution. However, sensory evaluation revealed that Sample-High exhibited a richer and more balanced retronasal aroma, whereas Sample-Low showed brief intensity in certain notes but lacked overall fullness. This discrepancy between prediction and observation suggested that the retronasal aroma difference could not be fully explained by a simple linear summation of individual compound DoT values. Previous studies have shown that aroma perception in alcoholic beverages arises from complex mixtures of odorants, and perceptual interactions such as masking, addition, or other non-linear effects may influence the final sensory expression [34,35,36].

**Figure 5:**
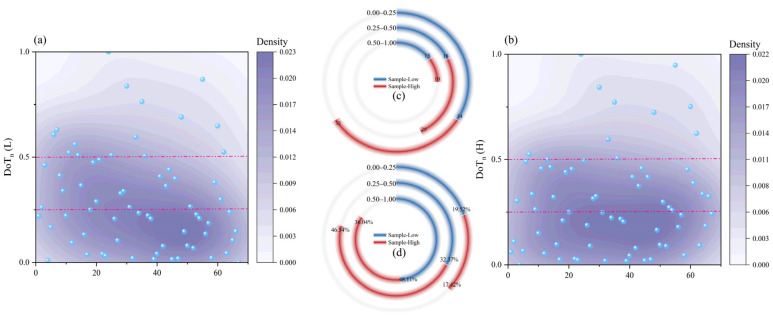
Differences in DoTn distribution characteristics of aroma compounds between the high- and low-alcohol systems. (**a**,**b**) show the DoTn distribution densities of compounds in the low- and high-alcohol systems, respectively. (**c**,**d**) show the differences in the number of compounds and their relative contribution proportions across different DoTn intervals in the two systems, respectively.

By applying min–max normalization to the DoT_log_ values in each system, we obtained DoT_n_, which reflected the relative contribution pattern of each compound within each system. This metric was used to first examine the within-system distribution pattern of contribution levels and then compare the distributional features between Sample-Low and Sample-High, with the final interpretation made in combination with the overall trend of ΔDoT_log_. Thus, DoT_n_ was used mainly to characterize distributional features rather than to directly represent absolute DoT-related magnitudes between the two systems. As shown in Figure 5a,b, the DoT_n_ density distribution of these compounds differed between the two systems. To compare their potential aroma contribution distributions more clearly, we conducted a segmented DoT_n_ analysis based on compound abundance and contribution proportion (Figure 5c,d). The results showed that within the 0.50–1.00 interval, Sample-Low contained 15 compounds, which was higher than the 10 compounds in Sample-High. In addition, the DoT_n_ weight proportion of Sample-Low in this interval reached 48.11%, exceeding the 36.04% in Sample-High. This may provide a plausible perceptual explanation for the lower retronasal aroma richness of Sample-Low. Previous work on odor-mixture processing indicates that when one component is much stronger than the others, it may cover the odor quality of the remaining components, a phenomenon described as complete overshadowing or masking [37,38,39]. Accordingly, the more concentrated contribution of a limited number of high-DoTn compounds in Sample-Low may have influenced the overall retronasal percept and reduced the perceptual expression of other aroma-active compounds, thereby weakening the overall aroma balance. In contrast, within the medium contribution interval (0.25–0.50), Sample-High contained 25 compounds and showed a DoT_n_ weight proportion of 46.54%, both of which were higher than the 18 compounds and 32.37% in Sample-Low. This distributional difference suggested that the retronasal aroma of Sample-High was supported by a broader group of medium contribution components, which could have reduced the chance of a small group of compounds becoming overly dominant and helped maintain a more balanced aroma profile. Within the low contribution interval (0.00–0.25), the two samples showed similar numbers of compounds with 34 in Sample-Low and 32 in Sample-High, and comparable weight proportions of 19.52% and 17.42%, respectively. These minor differences were likely to have exerted only a limited influence on the retronasal aroma distinctions between the two systems. Overall, the relatively high proportion of strongly contributing compounds and the limited presence of medium-contribution components in Sample-Low made its aroma system more prone to being strongly driven by a small number of compounds. In contrast, Sample-High displayed a more balanced contribution profile, which may help support greater retronasal aroma richness and harmony. Building on the understanding of the overall aroma contribution, the next step focused on the pronounced notes to analyze potential aroma compounds driving these sensory differences.

The floral and fruity note mainly originated from esters [40]. As shown in Figure 4a, the ROT values of esters varied significantly between the two ethanol systems, with the ratio of the high- and low-alcohol systems ranging from 1.04 to 4.02. In contrast with ethyl propionate, ethyl acetate, and ethyl lactate, which had ROT values above 10 μg/L, the ROT values of ethyl hexahydrobenzoate, ethyl 3-methylpentanoate, and ethyl 2-methylbutanoate were below 10 μg/L, which suggested that these esters were more easily perceived retronasally. Further analysis demonstrated that compounds with ROT ratios greater than 2 were predominantly distributed among those with lower ROT values. The ΔDoT_log_ results indicated that most compounds exhibited values below 0, theoretically suggesting that the floral and fruity notes were more pronounced in Sample-Low. Despite the differences in time–citation proportion during the E stage, the sensory evaluation demonstrated that Sample-High performed better overall across the intensity parameters. An examination of the DOT_n_ distribution further showed that Sample-Low contained more compounds within the high-contribution interval (0.50–1.00) and had a higher cumulative potential contribution of 53.19%. This pattern indicated that the floral and fruity note of Sample-Low was more likely to be dominated by a limited group of strongly contributing compounds, reflecting a tendency toward extreme-value driving. In contrast, Sample-High contained more compounds within the medium-contribution interval (0.25–0.50) and presented a larger potential contribution proportion of 51.10%. This distribution pattern suggested that the floral and fruity note of Sample-High was primarily shaped by the collective contributions of multiple medium-strength components. Within the low-contribution interval (0–0.25), the differences between the two samples were relatively small and likely exerted only a limited influence. Taken together, the results suggested that the aroma richness of the floral and fruity notes did not result from the dominance of a few strong contributors. Instead, it was driven by the balanced contributions of multiple components. It should be noted that the floral and fruity note in both ethanol system was short-lived, which was likely due to the rapid release of these compounds in the oral cavity and their low binding affinity to the mucosal surface [41]. In addition, its sensory expression may also be modulated, to some extent, by perceptual interactions among coexisting compounds. Previous research has shown that 1-propanol can weaken floral and fruity perception and suppress the volatilization of some ester compounds [42]. Other studies have further shown that long-chain fatty acids and their ethyl esters could modulate the retronasal persistence of fruity esters [43].

Pyrazines are typically associated with roasted notes. In this study, 2,6-dimethylpyrazine and 2,3,5-trimethylpyrazine were selected as representative compounds. Meanwhile, because furfuryl mercaptan exhibits sulfury, roasted, and burnt characteristics [44], it might have acted synergistically with pyrazines to enhance the roasted impression within the complex aroma matrix. According to the ROT analysis, both 2,6-dimethylpyrazine and 2,3,5-trimethylpyrazine exhibited ROT ratios above 2, suggesting that increasing ethanol concentration suppressed their release in the high-alcohol system, thereby reducing their accessibility to the olfactory system. In contrast, the ROT of furfuryl mercaptan was extremely low in both systems, with values of 0.0011 μg/L and 0.0007 μg/L, respectively, indicating that it could be perceived readily even at trace levels. All three compounds showed negative ΔDoT_log_ values, theoretically indicating a stronger roasted note in Sample-Low. However, the sensory results did not fully align with this prediction. Sample-Low only exhibited a rapid rise in the citation proportion of the roasted note during the E stage, which differed significantly from Sample-High. In contrast, Sample-High maintained a longer persistence of roasted characteristics during the F stage, and multiple intensity parameters confirmed that it demonstrated a more pronounced retronasal roasted characteristic across several sensory dimensions. To further clarify this difference, the DOT_n_ distribution showed that furfuryl mercaptan occupied an overwhelmingly dominant position within the 0.50–1.00 interval, with its potential contribution accounting for 95.80% in Sample-High and 94.66% in Sample-Low. The two pyrazine compounds were mainly distributed in the 0.00–0.25 interval, contributing only 4.20% and 5.34%, respectively. This distribution pattern indicated that the potential roasted contribution was driven primarily by compounds with high contribution levels within the current analytical framework. The overall trend supported the conclusion that Sample-High exhibited a more pronounced retronasal roasted characteristic. Notably, most pyrazines in Baijiu occurred at subthreshold levels, implying that their direct aroma contributions were limited, yet they might have participated in roasted note formation through synergistic enhancement [45]. Studies have also shown that the roasted note is not determined solely by the concentration of pyrazines, but rather arises from the complex interactions among key volatile compounds [46]. Therefore, the potential synergistic effects of multiple subthreshold pyrazines in Baijiu deserve further investigation in future studies.

Furans and lactones are characterized by distinctive caramel-like and fruity notes [47,48]. This study examined the caramel note by comparing the potent aroma contributions of furfural, 4-hydroxy-2,5-dimethylfuran-3-one, 4-hydroxy-2,3-dimethyl-2H-furan-5-one, and 5-ethyl-3-hydroxy-4-methyl-2(5H)-furanone between high- and low-alcohol systems. The ROT analysis showed that furfural had a markedly higher ROT value in the high-alcohol system, which suggested that increasing ethanol concentration exerted a certain suppressive effect on its olfactory perception. In contrast, the other three compounds exhibited consistently low and stable ROT values across both systems, indicating that they could be perceived at relatively low concentrations and were not substantially affected by ethanol level. According to the ΔDoT_log_ results, the theoretical aroma contribution trends of these compounds showed clear differentiation. Except for furfural, all compounds exhibited positive values, suggesting that Sample-High may have a theoretical advantage in aroma contribution. In agreement with the sensory observations, Sample-High showed a significantly higher time–citation proportion during the F stage than Sample-Low, displaying overall stronger perceptual intensity, slower attenuation, and greater aroma accumulation. Sample-Low showed only slight advantages in Plateau_90%_ and T_max_, indicating that Sample-High demonstrated a more persistent and stable olfactory performance for the caramel note. The distribution of compound DoT_n_ values further revealed differences in the contribution interval patterns between the two samples. Within the high-contribution interval (0.50–1.00), only Sample-Low contained a single compound, which accounted for 54.99%, suggesting that the caramel note was more susceptible to being strongly driven by high-contribution components. In contrast, Sample-High contained no compounds in this interval but included more components in the medium-contribution range (0.25–0.50) than Sample-Low, with a markedly higher cumulative potential contribution of 73.89%, compared with 37.27% in Sample-Low. This pattern indicated that the caramel note of Sample-High was primarily shaped by compounds with medium contribution levels. In the low-contribution interval (0–0.25), both samples contained the same number of compounds, although Sample-High exhibited a larger contribution proportion. Integrating the sensory observations with the potential contribution distribution indicated that caramel note perception of Sample-Low was likely dominated by a few high-contribution compounds, producing a rapid but relatively transient caramel impression. In Sample-High, the broader distribution of several medium-contribution components formed a more dispersed contribution pattern, which helped sustain a stronger and longer-lasting caramel perception.

Previous studies have reported that 3-methylnonane-2,4-dione possesses a strawlike aroma characteristic [49] and originates from furanoid fatty acids [50]. Based on its aroma characteristics, the hay note detected in the samples was likely attributed to this compound. ROT analysis showed that 3-methyl-2,4-nonanedione had low ROTs in both the high- and low-alcohol systems (2.44 and 1.91 μg/L, respectively), suggesting that it can be readily perceived. The negative ΔDoT_log_ value suggested that, theoretically, the aroma contribution of this compound was slightly greater in the low-alcohol system. From the sensory perspective, the citation proportion of the hay note in Sample-Low was temporarily and significantly higher than that in Sample-High during the middle of the E stage and the early phase of the F stage. This trend was consistent with the theoretical prediction. However, in the later phase of the E stage, the citation proportion of the hay note in Sample-High gradually increased, becaming significantly higher than that in Sample-Low during the middle and late phases of the F stage. All intensity parameters also supported the superior performance of Sample-High. The DoT_n_ value of this compound in Sample-High was slightly higher than that in Sample-Low, which suggested a certain advantage in its relative aroma contribution within the high-alcohol system. This observation aligned with the stronger hay note perception of Sample-High during the later phase of the E stage. On the whole, although increasing ethanol concentration might slightly suppress the monomeric activity of this compound, it would not necessarily diminish its relative contribution weight in the high-alcohol system. The low-alcohol system may promote a more intense and immediate aroma generated by 3-methylnonane-2,4-dione, whereas the high-alcohol system could enhance its persistence, resulting in a more stable and longer lasting retronasal perception.

Aldehydes are key contributors to the aroma profile of Baijiu. Short-chain saturated aldehydes (e.g., acetaldehyde) produce pungent and aldehydic notes, medium- to long-chain aldehydes (e.g., hexanal) impart grassy nuances, aromatic aldehydes (e.g., benzeneacetaldehyde) deliver honey- and sweet-like notes, and unsaturated aldehydes (e.g., (2E,6Z)-nona-2,6-dienal) contribute cucumber- and green-like aromas. Moreover, many long-chain saturated and unsaturated aldehydes exhibit fatty characteristics [20]. These observations indicated that chain length and saturation were critical structural factors influencing the aroma expression of aldehydes. From the sensory perspective, the time–citation proportion of the fatty note in Sample-Low was significantly higher than that in Sample-High during the later phase of the E stage, indicating a broader perception plateau and greater persistence. Sample-High showed an advantage only in I_max_, whereas its overall duration was comparatively weaker. To investigate the underlying chemical basis, several compounds associated with the fatty note were selected in this study, including octanal, nonanal, decanal, (2E)-2-nonenal, (2E)-2-dodecenal, and (2E,4E)-2,4-decadienal [20]. ROT analysis demonstrated that these aldehydes generally had low ROT values, which further declined in the low-alcohol system, yielding ratios between the two systems ranging from 1.10 to 2.37. Among them, (2E)-2-nonenal and (2E)-2-dodecenal showed the most pronounced reductions, which suggested that the volatility of medium- to long-chain unsaturated aldehydes might be strongly suppressed in the high-alcohol system. In contrast, long-chain saturated and polyunsaturated aldehydes showed only minor variations, which indicated lower sensitivity to alcohol concentration. In terms of total DoT, the combined proportion of these compounds in Sample-High reached 1.18%, which was notably higher than that in Sample-Low (0.47%). This theoretically suggested that Sample-High possessed a more pronounced fatty characteristic. However, this trend did not fully align with the actual sensory results. Analysis of the DoT_n_ distribution showed that both samples contained one compound in the high-contribution interval (0.50–1.00), with comparable contribution proportions, although Sample-Low showed a slightly higher value than Sample-High (39.37% vs. 38.24%). In the medium-contribution interval (0.25–0.50), Sample-High contained more compounds and exhibited a substantially higher cumulative contribution (39.15% vs. 24.67%), which might have contributed to its greater peak intensity. Conversely, Sample-Low showed a higher contribution proportion in the low-contribution interval (0.00–0.25), accounting for 35.95%, compared with 22.60% in Sample-High. Overall, the potential contribution pattern in Sample-Low was more broadly distributed, with aroma contributions spread more evenly across intervals. This distribution trend corresponded well with its broader and more persistent perceptual plateau observed in the sensory evaluation.

Phenols are typically associated with smoky characteristics, and previous studies have identified 2-methoxyphenol as a key compound responsible for the grain-like note [51]. In this study, 3-methylphenol, 4-methylphenol, 2-methoxyphenol, and 4-ethylphenol were selected as representative compounds to investigate their potential contributions to smoky or grain notes under different alcohol systems. ROT analysis showed that, except for 3-methylphenol, the remaining compounds generally had low ROT values. Among those, 2-methoxyphenol was scarcely affected by alcohol concentration, suggesting strong stability within the system, whereas 4-methylphenol and 3-methylphenol exhibited relatively high ROT ratios, implying that their release and perception were more susceptible to modulation by alcohol concentration. Based on the ΔDoT_log_ results, all compounds except 2-methoxyphenol showed negative values, which suggested that 3-methylphenol, 4-methylphenol, and 4-ethylphenol might enhance the smoky note of Sample-Low. In contrast, 2-methoxyphenol might contribute to a stronger grain note in Sample-High. Previous studies have reported that 2-methoxyphenol could be readily adsorbed onto the oral mucosa and remain there for an extended period, thereby contributing significantly to aroma persistence [41]. This unique retention property provides a chemical basis for its ability to sustain aroma longevity within the system. Based on the DoT_n_ distribution characteristics, the compounds associated with the smoky note in Sample-Low were mainly distributed within the 0.00–0.50 range, with the 0.25–0.50 interval contributing 52.77%. This indicated a more dispersed contribution pattern, which corresponded well with its stronger persistence and greater aroma accumulation observed in the sensory evaluation. In contrast, all contributions in Sample-High were confined to the 0.00–0.25 interval (100%), forming a highly concentrated low-contribution profile. Although this pattern supported a higher citation proportion during the early E stage, Sample-High exhibited markedly weaker persistence and lower aroma accumulation compared with Sample-Low. For the grain note, 2-methoxyphenol remained within the low-contribution interval in both systems. Analysis of the DoT_n_ differences further revealed a positive value, indicating a greater relative contribution weight in the high-alcohol system. This suggested that 2-methoxyphenol might play a more prominent role in Sample-High, consistent with its superior performance in the corresponding intensity parameters.

This study was intended to characterize retronasal aroma differences between representative high- and low-alcohol Jiangxiangxing Baijiu samples. However, because the present design did not include multiple independent samples within each alcohol level, the observed differences should be interpreted as differences between representative products associated with alcohol-level variation under the tested conditions, rather than as strictly controlled single-factor effects. Future studies using a broader set of independent samples or controlled dilution-based designs are needed to further validate these findings.

## 4. Conclusions

This study comprehensively investigated the dynamic retronasal aroma differences between high- and low-alcohol *Jiangxiangxing* Baijiu from two dimensions: time–citation proportion and time–intensity. The results showed that Sample-High exhibited greater retronasal aroma richness than Sample-Low. Significant differences in citation proportions were mainly observed for roasted, floral and fruity, smoky, hay, fatty, caramel, and grain notes, and the intensity-related results further indicated that roasted, grain, floral and fruity, hay, and caramel notes were more prominent in Sample-High, whereas fatty and smoky notes were more pronounced in Sample-Low. Overall, these sensory results reflected the relatively weaker retronasal aroma richness of Sample-Low. At the chemical level, this study found that the concentrations of 67 compounds did not differ significantly between the samples, which suggested that differences in compound concentration alone were unlikely to fully account for the retronasal aroma differences observed between the two samples. In contrast, most compounds showed lower ROTs in the low-alcohol system, and several compounds therefore exhibited larger DoTs under this condition. Further analysis of the DoTn distribution showed that, for most differential notes, Sample-Low contained a higher proportion of high-contribution compounds, making its aroma profile more susceptible to being driven by a small number of dominant components. In contrast, Sample-High presented a more balanced contribution structure, which was associated with superior retronasal aroma performance. Collectively, this study suggests that alcohol-level differences were closely associated with retronasal aroma perception in representative Jiangxiangxing Baijiu samples, and provides new insight into the sensory and perceptual differences between high- and low-alcohol products. At the same time, as the present comparison was based on representative samples from two common alcohol-level categories in the Baijiu market, the findings should be interpreted with appropriate caution. However, the overall trends were broadly consistent with previous reports concerning alcohol-level effects on aroma perception. Future studies using a wider range of alcohol-level gradients may provide further insight into how alcohol level shapes retronasal aroma perception.

## Figures and Tables

**Table 1:** ROT and DoT for aroma compounds of different classes under high- and low-alcohol systems.

Compounds ^a^	Class	C (L) ^b^	C (H) ^c^	ROT (H) μg/L ^d^	ROT (L) μg/L ^e^	Retronasal Aroma Descriptor	DoT (L) ^f^	DoT (H) ^g^	*p* ^h^	DoT_n_ (L) ^i^	DoT_n_ (H) ^j^
1,1-Diethoxyethane	Acetals	149,452.39 ± 11,411.11	435,348.42 ± 34,132.36	883.09	223.67	fruit, cream	668.19	492.98	**	0.60	0.60
Decanoic acid ^a^	Acids	1425.82 ± 184.14	1074.84 ± 60.99	31,327.90	6594.08	rancid, fat	0.22	0.03	**	0.01	0.00
Butanoic acid ^a^	Acids	46,685.81 ± 101.04	38,312.72 ± 1529.98	9875.25	4256.91	rancid, cheese, sweat	10.97	3.88	***	0.22	0.15
Acetic acid	Acids	2,409,795.19 ± 153,437.86	2,063,417.79 ± 13,878.84	253,501.50	157,897.33	sour, pungent	15.26	8.14	**	0.25	0.21
4-Methylpentanoic acid	Acids	600.66 ± 38.69	441.44 ± 7.97	1438.68	1116.50	pungent, cheese	0.54	0.31	**	0.03	0.02
Propanoic acid	Acids	50,268.83 ± 1066.78	74,224.91 ± 142.52	9722.62	5397.24	cheesy, vinegar	9.31	7.63	**	0.21	0.20
3-Methylbutanoic acid	Acids	118,549.15 ± 6248.04	127,666.2 ± 2249.81	2626.34	2217.93	sweat, rancid	53.45	48.61	*	0.36	0.37
2-Heptanol	Alcohols	2326.4 ± 179.37	2923.97 ± 118.95	104.87	42.01	fresh lemon, green	55.38	27.88	**	0.37	0.32
1-Octen-3-ol	Alcohols	171.74 ± 13.45	107.89 ± 6.57	59.49	47.91	mushroom, earthy, green	3.58	1.81	**	0.13	0.10
2-Nonanol ^a^	Alcohols	131.47 ± 5.24	165.51 ± 12.85	440.99	191.29	cucumber, waxy	0.69	0.38	***	0.04	0.03
2-Methyl-1-butanol	Alcohols	92,689.18 ± 212.39	105,362.93 ± 3273.49	22,386.71	21,246.19	alcoholic, greasy, whiskey	4.36	4.71	*	0.15	0.16
2-Phenylethanol	Alcohols	6377.23 ± 119.55	10,561.51 ± 611.56	646.40	543.83	floral, rose	11.73	16.34	**	0.23	0.27
1-Propanol	Alcohols	931,570.55 ± 59,199.51	2,397,456.14 ± 76,087.82	401,520.03	387,082.17	alcoholic, fusel	2.41	5.97	***	0.10	0.18
Nonanal ^a^	Aldehydes	74.04 ± 3.15	84.62 ± 13.48	52.53	31.10	citrus, green, fatty	2.38	1.61	*	0.10	0.09
(2E,4E)-2,4-Decadienal ^a^	Aldehydes	22.50 ± 0.20	22.86 ± 0.11	0.89	0.61	fried, wax, fat	36.74	25.77	***	0.33	0.31
3-Methylbutanal	Aldehydes	19,398.22 ± 244.48	27,313.17 ± 649.54	4.28	2.15	ethereal, aldehydic	9028.69	6378.09	***	0.84	0.84
Benzeneacetaldehyde	Aldehydes	2550.34 ± 153.62	3045.70 ± 182.35	243.63	144.97	honey, sweet	17.59	12.50	**	0.26	0.25
(2E)-2-Dodecenal ^a^	Aldehydes	17.52 ± 1.52	27.04 ± 3.44	44.20	24.57	green, fat, sweet	0.71	0.61	-	0.04	0.04
Hexanal ^a^	Aldehydes	2038.49 ± 16.79	2320.17 ± 1.05	19.82	16.29	grass, green	125.12	117.05	**	0.44	0.46
Vanillin	Aldehydes	35.48 ± 0.37	44.95 ± 0.30	134.09	104.06	vanilla, sweet, creamy	0.34	0.34	-	0.02	0.02
(2E,6Z)-nona-2,6-dienal ^a^	Aldehydes	127.46 ± 11.37	142.19 ± 5.90	0.08	0.07	cucumber, wax, green	1857.81	1856.79	-	0.69	0.72
Acetaldehyde	Aldehydes	458,591.06 ± 34,549.68	950,408.08 ± 21,672.7	43,756.79	25,262.99	pungent, ether, aldehydic	18.15	21.72	*	0.27	0.30
Octanal ^a^	Aldehydes	37.33 ± 6.49	64.81 ± 9.07	5.48	4.74	fatty, soapy, green	7.19	10.99	**	0.19	0.24
(2E)-2-Nonenal ^a^	Aldehydes	37.55 ± 1.19	191.16 ± 40.71	0.29	0.12	tallowy, fatty, oily	308.28	662.69	*	0.52	0.62
Propanal ^a^	Aldehydes	2264.21 ± 191.67	5257.72 ± 52.92	3149.05	2982.54	solvent, pungent	0.76	1.67	***	0.04	0.09
Decanal ^a^	Aldehydes	129.96 ± 16.12	935.1 ± 46.88	32.35	29.52	soap, orange peel, tallow	4.40	28.90	**	0.15	0.32
Ethyl octanoate	Esters	12,210.55 ± 699.37	6138.81 ± 340.49	265.85	77.75	fruity, wine, waxy	157.05	23.09	***	0.46	0.30
Ethyl 4-methylpentanoate	Esters	1344.25 ± 67.00	857.04 ± 54.06	5.14	1.72	fruit	759.39	166.64	***	0.61	0.49
Ethyl hexanoate	Esters	294,241.98 ± 9033.42	134,574.16 ± 8287.64	568.21	303.92	apple peel, sweet, pineapple	968.15	236.84	***	0.63	0.53
Isoamyl acetate	Esters	2060.18 ± 90.02	2638.53 ± 154.39	81.51	22.38	sweet, fruity, banana	92.04	32.37	***	0.41	0.34
Ethyl 2-methylpentanoate ^a^	Esters	119.31 ± 1.30	56.92 ± 3.05	3.85	2.85	fruity, apple, pineapple	41.81	14.78	***	0.34	0.26
Ethyl 3-methylpentanoate ^a^	Esters	23.22 ± 1.56	36.15 ± 2.14	0.31	0.08	pineapple, fruity	301.05	116.48	***	0.52	0.46
Ethyl valerate	Esters	11,057.26 ± 359.02	7149.27 ± 463.12	73.28	60.86	yeast, fruit	181.70	97.57	***	0.47	0.44
Ethyl benzoate	Esters	743.03 ± 42.15	1350.99 ± 91.42	105.37	31.39	fruity, wintergreen, green	23.67	12.82	***	0.29	0.25
Ethyl hexahydrobenzoate ^a^	Esters	10.14 ± 0.67	11.93 ± 0.06	0.10	0.05	fruity, cheese, winey	208.69	113.67	**	0.49	0.45
Ethyl butanoate	Esters	20,515.93 ± 44.94	27,966.09 ± 717.59	160.58	77.61	fruity, sweet, ethereal	264.35	174.15	***	0.51	0.50
Ethyl propionate ^a^	Esters	42,989.38 ± 2008.04	65,936.26 ± 1695.25	2262.08	1040.67	sweet, fruity, rum	41.31	29.15	**	0.34	0.33
Ethyl isobutyrate	Esters	20,028.77 ± 422.82	22,663.58 ± 1108.25	7.37	4.91	sweet, ethereal, fruity	4080.62	3075.88	**	0.76	0.77
Ethyl heptanoate	Esters	11,844.43 ± 893.35	10,927.09 ± 125.92	56.92	47.40	fruity, pineapple, cognac	249.89	191.96	*	0.50	0.50
Ethyl cinnamate	Esters	1193.60 ± 2.42	1621.04 ± 2.00	182.50	108.05	honey, cinnamon	11.05	8.88	***	0.22	0.22
Ethyl 3-phenylpropanoate	Esters	1917.16 ± 120.82	1886.19 ± 114.35	24.49	20.93	hyacinth, rose, honey	88.29	77.01	-	0.41	0.42
Ethyl phenylacetate	Esters	451.70 ± 28.5	837.00 ± 1.92	527.59	345.77	fruit, sweet	1.31	1.59	*	0.07	0.09
Ethyl 2-methylbutanoate	Esters	9614.45 ± 330.95	16,099.75 ± 1101.4	0.81	0.75	apple, green	12,829.86	19,045.46	***	0.87	0.95
Ethyl lactate	Esters	765,379.29 ± 45,931.29	1,888,738.75 ± 118,713.16	337,505.73	203,269.09	sharp, fruity, buttery	3.77	5.60	***	0.14	0.18
Ethyl 3-methylbutanoate	Esters	3681.74 ± 46.98	9298.84 ± 543.61	3.75	3.12	sweet, apple, pineapple	1178.83	2479.18	**	0.65	0.75
Ethyl acetate	Esters	1,039,920.63 ± 24,334.54	2,312,802.78 ± 48,024.14	40,873.88	39,300.22	pineapple, fruity, sweet	26.46	56.58	***	0.30	0.39
Furfural	Furans and Lactones	141,564.18 ± 12,358.4	195,686.68 ± 11,173.81	1079.97	310.94	sweet, woody, baked bread	455.28	181.20	**	0.56	0.50
γ-Nonanolactone ^a^	Furans and Lactones	18.33 ± 3.31	53.85 ± 10.05	43.10	11.97	coconut, peach	1.53	1.36	-	0.08	0.08
4-Hydroxy-2,5-dimethylfuran-3-one ^a^	Furans and Lactones	7.94 ± 0.62	11.38 ± 0.62	6.34	5.02	sweet, candy, caramel	1.58	1.74	-	0.08	0.09
4-Hydroxy-2,3-dimethyl-2H-furan-5-one ^a^	Furans and Lactones	795.24 ± 123.49	1609.56 ± 189.61	13.74	11.26	caramel, sugar, herb	64.52	110.56	**	0.38	0.45
4-Undecanolide ^a^	Furans and Lactones	294.89 ± 8.52	791.34 ± 23.49	24.26	21.95	peach, creamy, fatty	13.43	32.62	***	0.24	0.34
5-Ethyl-3-hydroxy-4-methyl-2(5H)-furanone ^a^	Furans and Lactones	0.12 ± 0.06	23.65 ± 2.91	2.03	1.14	caramel, sugar, herb	0.11	11.68	**	0.00	0.24
Acetophenone	Ketones	523.44 ± 25.55	364.66 ± 27.63	346.17	92.63	flower, almond, sweet	5.65	1.05	***	0.17	0.07
Acetoin	Ketones	7511.67 ± 7.59	15,714 ± 829.2	2514.87	810.28	sweet, buttery, creamy	9.27	6.25	**	0.21	0.19
3-Methylnonane-2,4-dione ^a^	Ketones	0.57 ± 0.15	0.61 ± 0.10	2.44	1.91	hay, caramel, burnt	0.30	0.25	-	0.01	0.02
4-Methylphenol	Phenols	309.49 ± 10.21	75.66 ± 3.42	80.94	28.86	medicine, phenol, smoke	10.72	0.93	***	0.22	0.06
3-Methylphenol ^a^	Phenols	3364.86 ± 253.56	1048.36 ± 21.06	466.79	185.69	fecal, plastic	17.39	2.25	***	0.26	0.11
4-Ethylphenol	Phenols	9.22 ± 0.19	9.59 ± 0.38	34.75	31.71	phenol, spice, musk	0.29	0.28	-	0.01	0.02
2-Methoxyphenol	Phenols	28.01 ± 1.64	54.74 ± 0.86	86.56	74.77	smoke, sweet, medicine	0.37	0.63	***	0.02	0.04
2,6-Dimethylpyrazine	Pyrazines	618 ± 52.27	649.75 ± 39.72	1934.71	954.76	nut, cocoa, roast beef	0.65	0.34	**	0.04	0.02
2,3,5-Trimethylpyrazine	Pyrazines	694.95 ± 9.93	1390.96 ± 88.58	5339.62	1920.19	nutty, roasted, musty	0.36	0.26	**	0.02	0.02
2-Methyl-3-furanthiol	Sulfides	4.74 ± 0.01	2.41 ± 0.11	0.02	0.02	sulfury, meaty, fish	263.95	125.40	***	0.51	0.46
Furfuryl mercaptan	Sulfides	36.79 ± 1.69	36.49 ± 1.25	0.0011	0.0007	sulfury, roasted, burnt	52,096.09	32,573.59	***	1.00	1.00
Dimethyl trisulfide	Sulfides	3.20 ± 0.26	4.09 ± 0.14	0.05	0.04	sulfur, fish, cabbage	79.11	76.80	-	0.40	0.42
β-Damascenone ^a^	Terpenes	1.24 ± 0.03	1.66 ± 0.04	2.10	0.62	apple, rose, honey	1.99	0.79	***	0.09	0.05
Linalool	Terpenes	104.03 ± 18.64	89.59 ± 0.69	9.49	8.22	flower, wood	12.66	9.44	-	0.23	0.22
Geosmin	Terpenes	102.34 ± 7.35	376.28 ± 22.72	26.69	10.72	musty, earthy, soil	9.55	14.10	**	0.21	0.26

Annotation: ^a^ Compounds, compounds marked with “a” were supplementally quantified, while those unmarked were referenced from previous studies [10,20]. ^b^ C (L), average concentration of triplicates of Sample-Low. ^c^ C (H), average concentration of triplicates of Sample-High. The letters ^d^ and ^g^ represent the retronasal olfactory threshold and dose-over-threshold in 50% vol ethanol aqueous solution, respectively. The letters ^e^ and ^f^ represent the retronasal olfactory threshold and dose-over-threshold in 40% vol ethanol aqueous solution, respectively. ^h^ *p*, *, **, and *** indicate DoT significance at *p* < 0.05, *p* < 0.01, and *p* < 0.001, respectively. The letters ^i^ and ^j^ represent the normalized distribution of the logarithmic DoT values of the compounds in low-alcohol and high-alcohol systems, respectively.

## Data Availability

The original contributions presented in this study are included in the article/Supplementary Materials. Further inquiries can be directed to the corresponding authors.

## Supplementary Materials

The following supporting information can be downloaded at https://www.mdpi.com/article/10.3390/foods15071156/s1, Table S1: List of food-grade standard compounds; Table S2: Different aroma descriptors and references; Table S3: Compounds and analytical parameters of GC-MS/MS.

## Abbreviations

The following abbreviations are used in this manuscript:

ROT	Retronasal olfactory threshold
DoT	Dose-over-threshold
RDM-TCATA	Retronasal dual-mode temporal check-all-that-apply
RDA-TI	Retronasal dual-attribute time–intensity
