# Supplementary material for: Characterization of Retronasal Aroma Differences in High- and Low-Alcohol Jiangxiangxing Baijiu Using Temporal Sensory Evaluation, Retronasal Olfactory Thresholds, and Dose-over-Threshold Analysis
Source: Foods. 2026 Mar 29;15(7):1156. doi: 10.3390/foods15071156 (PMC13072752; doi:10.3390/foods15071156)
Supplement: Supplementary file 1 [file foods-15-01156-s001.zip › foods-4205933-supplementary.pdf]

Table S1. List of Food-Grade Standard Compounds

CAS	Compounds [a]	Class	Purity
105-57-7	1,1-Diethoxyethane	Acetals	98.00%
334-48-5	Decanoic acid	Acids	98.00%
79-09-4	Propanoic acid	Acids	99.63%
503-74-2	3-Methylbutanoic acid	Acids	98.00%
64-19-7	Acetic acid	Acids	99.50%
107-92-6	Butanoic acid	Acids	99.00%
646-07-1	4-Methylpentanoic acid	Acids	98.00%
628-99-9	2-Nonanol	Alcohols	99.00%
71-23-8	1-Propanol	Alcohols	97.00%
137-32-6	2-Methyl-1-butanol	Alcohols	99.00%
543-49-7	2-Heptanol	Alcohols	98.00%
60-12-8	2-Phenylethanol	Alcohols	99.70%
3391-86-4	1-Octen-3-ol	Alcohols	99.54%
75-07-0	Acetaldehyde	Aldehydes	93.00%
123-38-6	Propanal	Aldehydes	98.52%
18829-56-6	(2E)-2-Nonenal	Aldehydes	98.00%
25152-84-5	(2E,4E)-2,4-Decadienal	Aldehydes	98.00%
122-78-1	Benzeneacetaldehyde	Aldehydes	97.00%
124-19-6	Nonanal	Aldehydes	95.00%
66-25-1	Hexanal	Aldehydes	100.00%
590-86-3	3-Methylbutanal	Aldehydes	96.60%
124-13-0	Octanal	Aldehydes	98.40%
112-31-2	Decanal	Aldehydes	99.00%
557-48-2	(2E,6Z)-nona-2,6-dienal	Aldehydes	99.00%
20407-84-5	(2E)-2-Dodecenal	Aldehydes	98.00%
121-33-5	Vanillin	Aldehydes	99.00%
2021-28-5	Ethyl 3-phenylpropanoate	Esters	99.61%
25415-67-2	Ethyl 4-methylpentanoate	Esters	99.89%
93-89-0	Ethyl benzoate	Esters	98.00%
103-36-6	Ethyl cinnamate	Esters	98.00%
105-37-3	Ethyl propionate	Esters	98.00%
108-64-5	Ethyl 3-methylbutanoate	Esters	99.00%
106-30-9	Ethyl heptanoate	Esters	98.00%
97-62-1	Ethyl isobutyrate	Esters	98.00%
141-78-6	Ethyl acetate	Esters	98.00%
106-32-1	Ethyl octanoate	Esters	99.00%
101-97-3	Ethyl phenylacetate	Esters	98.60%
123-66-0	Ethyl hexanoate	Esters	98.00%
97-64-3	Ethyl lactate	Esters	98.00%
3289-28-9	Ethyl hexahydrobenzoate	Esters	98.00%
105-54-4	Ethyl butanoate	Esters	99.00%

539-82-2	Ethyl valerate	Esters	95.00%
7452-79-1	Ethyl 2-methylbutanoate	Esters	99.00%
123-92-2	Isoamyl acetate	Esters	98.00%
5870-68-8	Ethyl 3-methylpentanoate	Esters	97.83%
39255-32-8	Ethyl 2-methylpentanoate	Esters	97.20%
3658-77-3	4-Hydroxy-2,5-dimethylfuran-3-one	Furans and Lactones	99.00%
28664-35-9	4-Hydroxy-2,3-dimethyl-2H-furan-5-one	Furans and Lactones	98.00%
698-10-2	5-Ethyl-3-hydroxy-4-methyl-2(5H)-furanone	Furans and Lactones	98.00%
104-61-0	γ-Nonanolactone	Furans and Lactones	97.00%
98-01-1	Furfural	Furans and Lactones	98.56%
104-67-6	4-Undecanolide	Furans and Lactones	99.71%
113486-29-6	3-Methylnonane-2,4-dione	Ketones	99.00%
513-86-0	Acetoin	Ketones	99.55%
98-86-2	Acetophenone	Ketones	99.82%
108-39-4	3-Methylphenol	Phenols	98.00%
90-05-1	2-Methoxyphenol	Phenols	99.00%
106-44-5	4-Methylphenol	Phenols	99.00%
123-07-9	4-Ethylphenol	Phenols	95.00%
108-50-9	2,6-Dimethylpyrazine	Pyrazines	98.00%
14667-55-1	2,3,5-Trimethylpyrazine	Pyrazines	99.12%
3658-80-8	Dimethyl trisulfide	Sulfides	98.00%
28588-74-1	2-Methyl-3-furanthiol	Sulfides	99.22%
98-02-2	Furfuryl mercaptan	Sulfides	99.29%
78-70-6	Linalool	Terpenes	99.10%
19700-21-1	Geosmin	Terpenes	99.32%
23726-93-4	β-Damascenone	Terpenes	98.94%

[a]Compounds, they were sourced from Sigma-Aldrich Co., Ltd. (Shanghai, China), Shanghai Titan Scientific Co., Ltd. (Shanghai, China), Anhui JinDragon Flavor & Fragrance Co., Ltd. (Chuzhou, China), and Wuhan Lullaby Pharmaceutical Chemical Co., Ltd. (Wuhan, China).

Table S2. Different aroma descriptors and references.

Aroma descriptors	References[a]
Floral and fruity	ethyl 3-phenylpropanoate, 2-phenylethanol, ethyl 3-methylbutanoate, isoamyl acetate, and ethyl hexanoate
Roasted	trimethylpyrazine, and tetramethylpyrazine
Caramel	4-hydroxy-2,3-dimethyl-2H-furan-5-one
Acidic	acetic acid, and propionic acid
Grassy	hexanal, and (2E,6Z)-2,6-nonadienal
Grain	steamed mixture of sorghum, rice, and wheat
Hay	dried rice straw, and rice hulls
Oxidized oil-like	(2E)-2-nonenal, and (2E,4E)-2,4-nonadienal
Smoky	2-methoxyphenol, 4-methylphenol, and 4-ethylguaiacol
Alcoholic	40% vol or 50% vol ethanol aqueous solution
Animal-like	4-ethylphenol, and 3-methylphenol
Distilled spent Grains	distilled spent grains of *Jiangxiangxing* Baijiu
Earthy-mouldy	geosmin
Sulfur	dimethyl trisulfide, and 2-methyl-3-furanthiol
Daqu-like	high-temperature Daqu

[a]References, mixed in 10% aqueous ethanol solution.

Table S3. Compounds and analytical parameters of GC-MS/MS

CAS	Compounds	RI [a]	RT [b]	Q [c]	CE（eV）[d]	q [e]	CE（eV）	Standard curve	R^2	Liner range μg/L	LOD [f]	LOQ [g]	Recovery (%)
104-61-0	γ-Nonanolactone	2018	37.494	138-95	10	138-123	10	y=3.74E-4 * x + 4.71E-5	0.9998	12.53-6416.04	3.35	11.17	108
3658-77-3	4-Hydroxy-2,5-dimethylfuran-3-one	2030	37.496	128-85	8	109-67	10	y= 2.69E-3 * x + 0.78E-6	0.9991	2.37-1216.00	0.03	0.09	88
28664-35-9	4-Hydroxy-2,3-dimethyl-2H-furan-5-one	2198	40.96	128-72	4	128-83	4	y=1.06E-5 * x + 7.27E-5	0.9990	51.54-1659.00	0.04	0.13	96
104-67-6	4-Undecanolide	2247	41.974	148-91	15	148-120	5	y = 8.40E-4 * x - 4.31E-5	0.9991	5.06-1297.44	0.91	3.03	102
698-10-2	5-Ethyl-3-hydroxy-4-methyl-2(5H)-furanone	2263	42.118	142-86	5	142-97	5	y=8.41E-5 * x + 5.861E-6	0.9869	0.60-1074.33	0.07	0.23	98

Annotation: [a]RI, retention Index; RT, retention time; [c]Q, quantifier transition; [d]CE, collision energy; [e]q, qualifier transition; [f]LOD, limits of detection. [g]LOQ, limits of quantitation.
